# Involvement of macrophage migration inhibitory factor and its receptor (CD74) in human breast cancer

**DOI:** 10.3892/or.2014.3272

**Published:** 2014-06-16

**Authors:** VINCENT RICHARD, NADÈGE KINDT, CHRISTINE DECAESTECKER, HANS-JOACHIM GABIUS, GUY LAURENT, JEAN-CHRISTOPHE NOËL, SVEN SAUSSEZ

**Affiliations:** 1 Laboratory of Anatomy and Cellular Biology, Faculty of Medicine and Pharmacy, University of Mons, B-7000 Mons, Belgium; 2 Department of Medical Oncology, CHU Ambroise Paré, B-7000 Mons, Belgium; 3 Laboratory of Image, Signal Processing and Acoustics, Ecole Polytechnique de Bruxelles, B-1050 Brussels, Belgium; 4 Institute of Physiological Chemistry, Faculty of Veterinary Medicine, Ludwig Maximilians University, D-80539 Munich, Germany; 5 Laboratory of Histology, Faculty of Medicine and Pharmacy, University of Mons, B-7000 Mons, Belgium; 6 Department of Pathology, Erasme Hospital, B-1070 Brussels, Belgium; 7 Department of Oto-Rhino-Laryngology, Faculty of Medicine, Free University of Brussels (ULB), CHU St-Pierre, B-1000 Brussels, Belgium

**Keywords:** breast cancer, MIF, CD74, microenvironment

## Abstract

Macrophage migration inhibitory factor (MIF) and its receptor CD74 appear to be involved in tumorigenesis. We evaluated, by immunohistochemical staining, the tissue expression and distribution of MIF and CD74 in serial sections of human invasive breast cancer tumor specimens. The serum MIF level was also determined in breast cancer patients. We showed a significant increase in serum MIF average levels in breast cancer patients compared to healthy individuals. MIF tissue expression, quantified by a modified Allred score, was strongly increased in carcinoma compared to tumor-free specimens, in the cancer cells and in the peritumoral stroma, with fibroblasts the most intensely stained. We did not find any significant correlation with histoprognostic factors, except for a significant inverse correlation between tumor size and MIF stromal positivity. CD74 staining was heterogeneous and significantly decreased in cancer cells but increased in the surrounding stroma, namely in lymphocytes, macrophages and vessel endothelium. There was no significant variation according to classical histoprognostic factors, except that CD74 stromal expression was significantly correlated with triple-negative receptor (TRN) status and the absence of estrogen receptors. In conclusion, our data support the concept of a functional role of MIF in human breast cancer. In addition to auto- and paracrine effects on cancer cells, MIF could contribute to shape the tumor microenvironment leading to immunomodulation and angiogenesis. Interfering with MIF effects in breast tumors in a therapeutic perspective remains an attractive but complex challenge. Level of co-expression of MIF and CD74 could be a surrogate marker for efficacy of anti-angiogenic drugs, particularly in TRN breast cancer tumor.

## Introduction

Breast cancer (BC) is the most frequently occurring malignant disease among women in the Western hemisphere, inflicting one in eight women (1). Despite marked progress in disease management, morbidity and mortality remain a public health concern, prompting efforts to advance our understanding of BC biology, with the aim of developing innovative approaches. In this respect, particular attention should be given to promoters of cell growth and microenvironment.

Macrophage migration inhibitory factor (MIF) is a pleiotropic inflammatory cytokine of 12.5-kDa monomeric molecular weight originally described as a T cell lymphokine modulating macrophage motility. Subsequently, MIF was shown to be produced by a variety of immune and non-immune cells such as B- and T-lymphocytes as well as endocrine, endothelial and epithelial cells of diverse histogenetic origin. Pathophysiologically, MIF plays a pivotal role in various autoimmune and inflammatory disorders, such as rheumatoid arthritis, systemic lupus erythematosus, septic shock and atherosclerosis (2,3). In addition, there is growing evidence that MIF is involved in cancerogenesis and progression. Currently, there is a general consensus that MIF promotes tumor growth by several mechanisms; it stimulates cancer cell proliferation by triggering the MAPK/PI3K/Akt pathways, inhibits induction of p53-dependent apoptosis, increases production of vascular endothelial growth factor (VEGF) and inhibits the antitumor immune response (4–6). Moreover, it modulates metastatic behavior of tumor cells and affects tumor stromal cells (7,8). On the cellular level, MIF is stored in the cytoplasmic compartment and is released in response to several stimuli. In breast cancer cells, signaling is triggered by its receptor CD74, then channeled via the Akt pathway, with the involvement of Src and PI3K (9). Additionally, CD44 can be recruited to the complex with CD74 and G-protein coupled receptors CXCR2 and CXCR4 can act as receptors, inducing rapid activation of integrins (10). Within the cell, c-Jun activation domain-binding protein-1 (JAB1) serves as binding partner, thereby reducing secretion and autocrine growth stimulation (11). MIF has also been reported to inhibit apoptosis by binding to p53 (12).

MIF serum levels are elevated in breast cancer patients (13) and MIF has been shown to be overexpressed in breast cancer tissue compared to normal breast. Correlations with classical histoprognostic factors remain controversial (14–16).

Looking at a receptor, CD74 is expressed in breast cancer tissue and its presence appears to be correlated with lymph node invasion and triple-negative tumors (17–19) making a correlation study attractive.

Due to the pro-tumoral activities, the MIF pathway might be considered as a potential therapeutic target. Of note, tumor-activated HSP90 chaperone complex protects MIF from degradation, suggesting that HSP90 inhibitors could serve as anti-MIF therapeutic agents. Indeed, the HSP90 inhibitor 17-N-allylamino-17-demethoxygeldanamycin (17-AAG) inhibits growth of MIF-expressing breast tumors in mice (20). Regarding the MIF receptor, milatuzumab, a humanized anti-CD74 antibody, has clinical activity on lymphomas and has been tested *in vitro* with some success as an antibody-drug conjugate on solid cancer cell lines positive for CD74 (21).

These considerations led us to an immunohistochemical assessment of expression of MIF and CD74 in serial sections of human breast cancer tumor specimens, mapping their profiles in cancer and stromal cells. In parallel, the serum level of MIF was determined in breast cancer patients.

## Materials and methods

### Breast cancer patients and healthy women

Formalin-fixed, paraffin-embedded, residual tissue material of diagnostic biopsies of 96 breast cancer tumors (Table I), which were available for retrospective analysis by immunohistochemistry, were examined for MIF expression and 59 of them for CD74. In each case, the pathological stage and histological grade were defined according to the criteria of the World Health Organization 2012. Estrogen receptor (ER), progesterone receptor (PR) status, Ki-67 labeling index and HER2 expression were evaluated at the time of the original diagnosis by immunohistochemistry, as previously described (22–24). Positivity for ER and PR as well as HER2 score has been defined previously (25). The characteristics of the tumors are outlined in Table I. Residual tumor-free breast tissue blocks from 16 breast plasties for esthetic purposes were used as reference specimens of healthy tissue.

Blood samples from 36 newly diagnosed early breast cancer female patients (BCP) were obtained prospectively for determining serum level of MIF, prior to any breast cancer treatment (Table II). Twenty-two healthy women (HW) were also enrolled in this prospective study as a control group. In both cohorts, 10 ml of blood were obtained, centrifuged at 4°C and sera stored at −20°C until assaying.

This study was approved by the Ethics Committee of Erasme Hospital, Brussels, Belgium, according to the international and Belgian laws (P2008/314 and A2013/016).

### Determination of MIF serum levels

Serum concentration was assayed by a sandwich enzyme-linked immunosorbent assay (ELISA) using a commercial kit (DuoSet ELISA Development kit, R&D Systems, Minneapolis, MN, USA). The assays were carried out according to the instructions provided by the supplier. MIF concentrations in serum samples were determined by interpolation from a reference curve established with increasing concentrations of recombinant human MIF.

### Immunohistochemistry on tissue specimens and assessment

For immunostaining of MIF, after antigen retrieval by microwave treatment, sections were pretreated with hydrogen peroxide to block endogenous peroxidase activity. Thereafter, they were exposed to casein to avoid false-positive staining. These steps were followed by sequential incubations with (i) primary antibody (rabbit polyclonal anti-human MIF (26), (ii) post-blocking (Immunologic, The Netherlands), (iii) poly-dextran secondary antibody against rabbit immunoglobulins. Immunocomplexes were finally visualized by exposure to the chromogen diaminobenzidine in the presence of H_2_O_2_. Sections were counterstained with luxol fast blue prior to light microscopy examination.

For immunostaining of CD74 (rabbit polyclonal anti-human CD74 FL-296, Santa Cruz Biotechnology, Santa Cruz, CA, USA), immunohistochemistry was performed using a procedure similar to that described previously for MIF (27).

MIF and CD74 expressions in the glandular and in the stromal compartments were assessed by light microscopy and quantified according to a modified Allred score (22).

### Statistical analysis

Non parametric analysis was carried out using the Mann-Whitney test and Spearman’s rank order correlations was used.

## Results

### MIF expression is increased in carcinoma cells and in the stroma of breast cancer tissue

**Figure 1:**
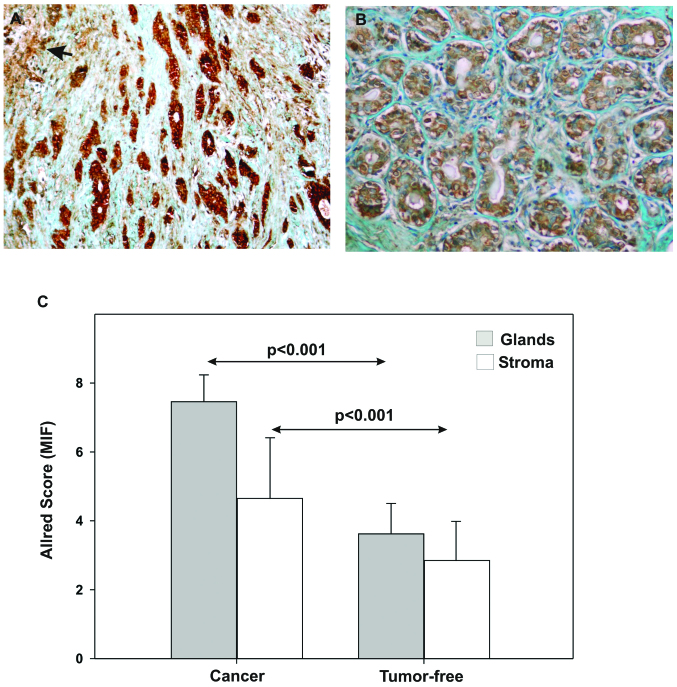
Immunohistochemical detection of MIF in non-cancerous breast (B) and breast cancer tissue sections with a strong positivity in the peritumoral fibroblasts (arrow) (A). Semi-quantitative analysis (Allred score) of MIF expression in glandular and stromal compartments (C) (Mann-Whitney test, p<0.001).

The intensity of immunohistochemical staining for MIF was assessed in tumor-free breast tissues (tumor-free, n=16; Fig. 1B) and in invasive ductal and lobular carcinomas (cancer, n= 96; Fig. 1A). The staining intensity, quantified using modified Allred scores, was markedly increased in carcinomas compared to tumor-free specimens (Mann-Whitney test, p<0.001; Fig. 1C). With regard to the distribution of the immunohistochemical signal, it was strong and homogeneous in carcinoma cells and appeared essentially extra-nuclear (i.e. cytoplasmic and membrane staining). By contrast, the signal intensity was weaker and its presentation more heterogeneous in the stromal compartment (Fig. 1A), although it showed the same extra-nuclear distribution. In the latter compartment, peritumoral fibroblasts were the most intensely stained, whereas lymphocytes and macrophages as well as endothelial cells showed weaker immunostaining.

**Figure 2:**
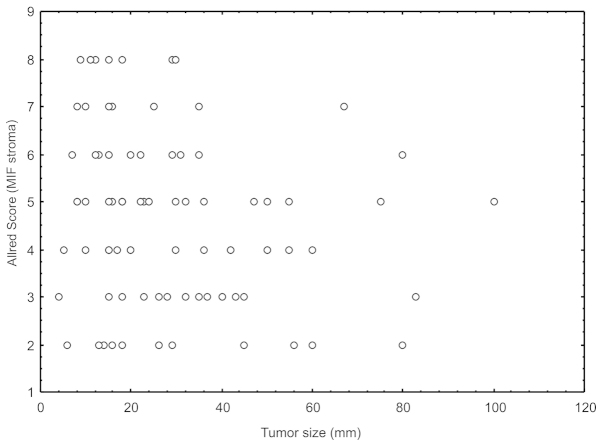
Significant inverse correlation between MIF stromal expression and BC tumor size (Spearman’s rank order correlations, p=0.02).

There was no significant correlation of staining with histological type, nodal status, histological grade, HER2 amplification, hormonal receptor status and the Ki-67 index (Mann-Whitney test NS, data not shown). However, we found a significant inverse correlation between tumor size and stromal positivity (Spearman’s rank test, R=−0,238, n=91, p=0.02; Fig. 2).

### Serum MIF levels are increased in breast cancer patients

**Figure 3:**
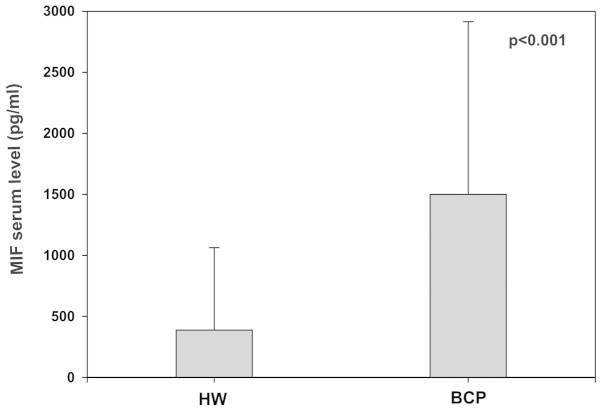
Serum level of MIF is increased in BCP compared to HW (Mann-Whitney test, p<0.001).

In addition to the immunohistochemical analysis of MIF expression in breast tumors, we used an ELISA to compare MIF levels in serum of 36 patients with BC (Table II) to those of 22 healthy individuals. Fig. 3 shows the average serum level for each group. In healthy individuals, we found a mean level of 387 pg/ml. In contrast, the mean concentration in patients reached 1,500 pg/ml, a concentration approximately four-fold higher than that recorded in healthy individuals (Mann-Whitney test, p<0.001; Fig. 3). We did not find any significant variation of the serum levels according to the tumor characteristics.

### CD74 expression in breast cancer tissues

**Figure 4:**
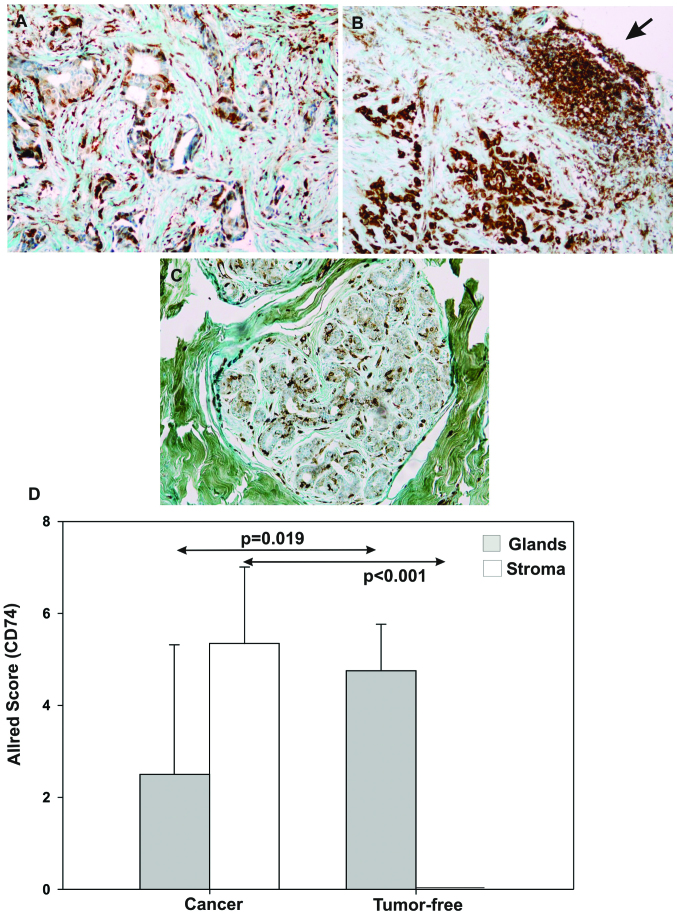
Immunohistochemical detection of CD74 in non-cancerous breast (C) and breast cancer tissue sections (A) with peritumoral lymphocytes (arrow, B). Semi-quantitative analysis (Allred score) of CD74 expression in glandular (Mann-Whitney test, p=0.019) and stromal (Mann-Whitney test, p<0.001) compartments (D).

CD74 expression was evaluated by immunohistochemistry in tumor specimens (cancer, n=59; Fig. 4A) and compared to that observed in tumor-free breast tissues (tumor-free, n=15; Fig. 4C). Compared with tumor-free tissues, immunostaining intensity analysis of cancer breast biopsies, quantified according to a modified Allred scoring, revealed a significant decrease in CD74 expression in the neoplastic glandular compartment (Mann-Whitney test, p=0.019; Fig. 4D), contrasting with an increased expression in the peritumoral stroma (Mann-Whitney test, p<0.001; Fig. 4D). In cancer cells, immunoreactivity for CD74 signal was weak and heterogeneous, mostly located on the membrane and in the cytoplasm. CD74 positivity was observed in the stroma surrounding carcinomas, namely in lymphocytes (arrow, Fig. 4B), macrophages and vessel endothelium, with a similar intracellular distribution.

**Figure 5:**
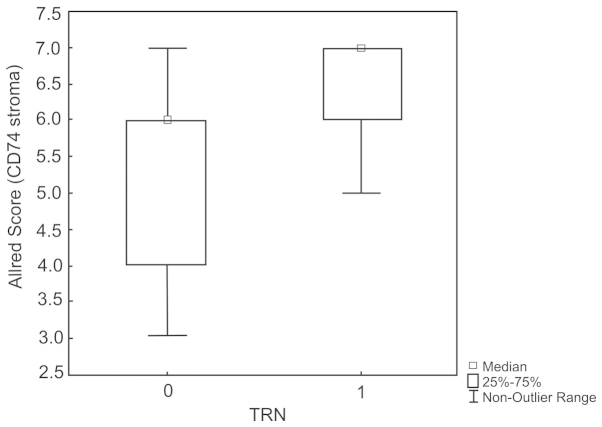
Stromal expression of CD74 correlates with the triple negative status of breast tumors (Mann-Whitney test, p=0.02).

There was no significant variation according to histological type, tumor size, nodal status, histological grade, HER2 amplification and Ki-67 index in either compartment. However, stromal CD74 expression appeared significantly correlated with triple-negative receptor (TRN) status and the absence of estrogen receptors (Mann-Whitney test, p=0.02; Fig. 5).

## Discussion

Numerous clinical and experimental data suggest that MIF can play a role in the pathogenesis of various solid tumors. We here combined serum level assessment with immunohistochemical detection. In this respect, our study is the first to report the semi-quantitative immunohistochemical evaluation of both expression of MIF and CD74 in serial sections of biopsy specimens, as well as their distribution between cancer cells and the peritumoral stroma. Our results allow us to consider suggestions on the involvement of this cytokine in breast tumor biology.

We confirmed the significant increase of MIF serum levels in BCP, observed previously by others (15,16). In a set of 98 serum proteins, MIF was able to discriminate normal tissue from breast cancer (28), although the authors considered the elevation of serum MIF more indicative of the inflammatory response to breast tumor than to the tumor itself. Correlations between MIF and classical histoprognostic factors remain controversial. A negative correlation with the number of involved nodes and a positive correlation with poor response to neoadjuvant chemotherapy have been reported (13,15). Recent data in mice suggest an immunosuppressive role of MIF favoring BC metastasis (29). Furthermore, this cytokine could also contribute to the systemic metabolic disturbances associated with BC (30). Thus, the increased serum level of MIF in BCP could be a non-specific signature of a systemic response to breast cancer with immune and metabolic implications.

Our data extended the data basis for an increased expression of MIF in breast cancer tumors (14–16). Of note, upregulation concerns tumor cells and peritumoral stroma. Verjans *et al* described a significant increase of MIF in breast carcinoma, this increase showing a positive correlation with the ER/PR status and a negative one with tumor size, in association with better overall survival. This data set intimated a beneficial role of intracellular MIF, whereas extracellular MIF is pro-oncogenic by promoting cancer cell-stroma interactions (14). Increased MIF positivity in cancer and stromal cells, including tumor-associated macrophages, correlated inversely with nodal involvement and also led to suggestions for a role of MIF in tumor-stroma interactions (15). We observed an inverse correlation between stromal MIF expression and tumor size, as well as an elevated MIF presence in fibroblasts surrounding the tumor tissue. This is consistent with the hypothesis that MIF could modulate the tumor size by inhibiting recruitment of cancer-associated fibroblasts (CAFs)/myofibroblasts, eventually resulting in retardation of tumor growth (7). These CAFs are known to be the source for effectors shaping a pro-tumoral microenvironment such as the chemokine CXCL-1 and interleukins-6 and -8 and may be involved in tumorigenesis (31,32).

Following profiling of MIF expression, we proceeded to map CD74 in breast cancer and in tumor-free tissue. We showed that the CD74 positivity is significantly increased in lymphocytes, macrophages and endothelial cells but heterogeneous in neoplastic cells. A correlation was detected between tumor stromal CD74 expression and the tumor triple receptor-negative status (TRN), including the absence of ER itself. A correlation between CD74 expression in BC, TRN status and lymph node invasion has previously been reported (17,18).

To date, co-expression of MIF and CD74 has not been studied in breast cancer but it has been described in prostate and non-small cell lung cancer. In prostate cancer, MIF was intense but CD74 staining was weak and patchy (33). In lung cancer, CD74 was mainly detected in the stromal compartment or in stromal and epithelial cells. Co-expression of MIF and CD74 was associated with greater vascularity and higher levels of pro-angiogenic CXC chemokines (34). We suggest that, in breast cancer, high-level expression of CD74 could be a marker of increased vascularity in stroma. Since bevacizumab, an anti-VEGF antibody, appears to be more efficient in TRN breast cancer (35), stromal CD74 expression could be examined as a predictive marker of response to bevacizumab-based therapy.

Our findings showed an increased extent of MIF expression in cancer cells and in stromal fibroblasts of BC tumor, in contrast to a less uniform increase of CD74 expression mainly in stromal lymphocytes, macrophages and endothelium. This could suggest a pro-oncogenic role of MIF in BC tumors taking place predominantly in the stromal compartment. MIF, secreted by tumor cells, could then modulate the immune microenvironment and its neovascularization, favoring escape from immune surveillance and tumor cell dissemination. Experimental data published show that MIF suppression in breast cancer cell lines does not affect their proliferation *in vitro* but causes delayed tumor growth in mice, increasing the prevalence of an immune suppressive myeloid-derived population within the tumor (28,36). Xu *et al* showed that MIF might promote angiogenesis in BC tumors (16). Finally, the discordance between the MIF and CD74 expression on cancer cells suggests that MIF could act on cells through other types of receptors, such as CXCR4 and CXCR7 (7,37). *In vitro* studies on MIF/CD74 (CD44) knockdown by siRNA approach, as reported for clear cell renal carcinoma (38), shed light on this aspect.

In conclusion, our data support the concept of a functional role of MIF in human breast cancer. In addition to auto- and paracrine effects on cancer cells, MIF could contribute to shape the microenvironment leading to immunomodulation and angiogenesis, these aspects deserving further investigations. Interfering with MIF effects in breast tumors in a therapeutic perspective remains an attractive but complex challenge, notably depending on the development of suitable MIF inhibitors. Level of co-expression of MIF and CD74 could be a surrogate marker for efficacy of anti-angiogenic drugs, particularly in TRN breast cancer tumors.

## Figures and Tables

**Table I:** Tumor characteristics.

	MIF data		CD74 data	
		
Variable	n (%) n=96	Level of sign.	n (%) n=59	Level of sign.
Tumor size		S		NS
T1	43 (45)		23 (39)	
T2	40 (42)		27 (45)	
T3	10 (11)		7 (12)	
T4	3 (3)		2 (3)	
Histological type		NS		NS
Invasive ductal	84 (87)		50 (85)	
Invasive lobular	11 (11)		9 (15)	
Other	1 (1)		0	
Lymph node status		NS		NS
N0	62 (65)		35 (59)	
N0–3	34 (35)		20 (34)	
NE			4	
Histological grade		NS		NS
G1–2	74 (77)		50 (85)	
G3	22 (23)		8 (15)	
NE			1	
Estrogen receptor status		NS		S
Negative	17 (18)		8 (14)	
Positive (>1%)	79 (82)		50 (85)	
NE	1			
Progesterone receptor status		NS		NS
Negative	25 (26)		12 (20)	
Positive (>1%)	71 (74)		46 (78)	
NE			1	
Triple receptor negative status		NS		S
Negative	83 (86)		52 (88)	
Positive (>1%)	13 (14)		6 (10)	
NE			1	
Ki-67		NS		NS
Low (≤15%)	47 (49)		29 (49)	
High (>15%)	49 (51)		29 (49)	
NE			1	
HER-2		NS		NS
Amplified	7 (7)		5 (8)	
Non amplified	89 (93)		53 (90)	
NE				1

NE, non evaluable; S, significant; NS, non significant.

**Table II:** Characteristics of patients/tumors for MIF serum measurements.

Characteristics	No.
Total no. of patients	36
Total no. of tumors	37
Mean age (years)	56 (range 30–80)
Tumor histological type	no./37
Invasive carcinoma NST	34
Lobular invasive carcinoma	2
Others	1
T1	22
T2	13
T3–T4	2
Positive node	19
Grade III	16
Ki-67 index >15%	12
Positive hormone receptor	29
Triple receptor negative	7
HER2 amplified	4
Neoadjuvant chemotherapy	17
